# Anatomy of the sacroiliac joints in children and adolescents by computed tomography

**DOI:** 10.1186/s12969-017-0210-0

**Published:** 2017-11-25

**Authors:** Anna Zejden, Anne Grethe Jurik

**Affiliations:** 1 Department of Radiology, Aarhus University Hospital, Noerrebrogade 44, 8000 Aarhus C, Denmark; 2 Department of Clinical Medicine, Aarhus University, Nordre Ringgade 1, 8000 Aarhus C, Denmark

**Keywords:** Sacroiliac joints, Computed tomography, Juvenile individuals, Anatomy, Sacroiliitis, Juvenile spondyloarthritis

## Abstract

**Background:**

Diagnosing sacroiliitis by magnetic resonance imaging (MRI) in children/adolescents can be difficult due to the growth-related changes. This study analyzed the normal osseous anatomy of the sacroiliac joints (SIJ) in a juvenile population using computed tomography (CT).

**Methods:**

The anatomy of the SIJ was retrospectively analyzed in 124 trauma patients aged 9 months – <18 years by CT, based on 2 mm slices in axial, semi-axial and semi-coronal planes. The following anatomical features were recorded: intersegmental fusion of the sacral vertebral segments 1–3 (S1-S3), ossified nuclei (antero-superior at S1, lateral to the intervertebral spaces and lateral to S1 and S2) and joint facet defects larger than 3 mm.

**Results:**

Fusion of S1/S2 started at the age of 6 years and was complete after the age of 13 years in most girls and after the age of 14 years in most boys. Fusion of S2/S3 started at the age of 9 years, but could remain incomplete up to 18 years in both genders. Ossified nuclei antero-lateral at S1 and/or in the joint space were observed until the age of 18 years and occurred in 77% of individuals ≥13 years with intraarticular localization in 64% of girls and 60% of boys. Joint facet defects >3 mm occurred in 21 children/adolescents (17%) located to both the iliac and sacral joint facets.

**Conclusions:**

Normal osseous SIJ structures in children and adolescents vary considerably. Attention to these normal anatomical structures during growth may help to avoid false positive findings by MRI.

## Background

The sacroiliac joints (SIJ) play an important role in spondyloarthritis as the diagnosis often is confirmed based on sacroiliitis by imaging. In adults the early diagnosis of sacroiliitis is currently based on magnetic resonance imaging (MRI) [1, 2]. Since 2009 bone marrow edema (BME), highly suggestive of sacroiliitis, has been part of the classifications criteria for adult SpA as defined by Assessment of SpondyloArthritis (ASAS) International Society [1]. Inflammatory changes in the sacroiliac joints is also frequent in juvenile spondyloarthritis (JSpA) [3, 4] but the classification or diagnostic criteria used in adults seems not to be applicable in juvenile patients [5]. One of the explanations for somewhat different sacroiliac joints changes in children/adolescents compared with adults is the persistence of growth related edematous changes and lack of a clear delineation of minor osseous structures by MRI; in juvenile patients this is often a big challenge for radiologists [6, 7]. Usually both active and chronic MRI findings are required to establish the diagnosis in children and adolescents and distinct knowledge about the complicated anatomy of SIJ is therefore important [8]. Computed tomography (CT) is superior to MRI for visualizing detailed osseous anatomy in addition to pathological structural lesions [9–11] and can display chronic osseous changes in sacroiliitis, but not signs of inflammatory activity [9, 12, 13]. However, due to exposure to ionizing radiation, CT is not commonly used as the diagnostic method for diagnosing SIJ changes, especially not in children and adolescents.

For both adults and children/adolescents, early and correct diagnosis of inflammatory changes in the SIJ is only possible with precise knowledge of the structural anatomy and developmental variation of SIJ. A literature review resulted in a few studies analyzing the anatomy of the SIJ based on CT in adults [10, 13–15]; two of these focused on prevalence and appearance of anatomical variants [14, 15]. In children and adolescents, CT evaluation was performed in one study with the purpose of evaluating developmental features. A total of 25 juvenile individuals were examined by single slice CT resulting in the detection of ossification centers in the SIJ in individuals aged >15 years [16].

The purpose of this study was to analyze the normal osseous anatomy of the SIJ in children and adolescents using current multislice CT technique.

## Methods

### Study population

The study was designed as a retrospective analysis conducted at the Department of Radiology, Aarhus University Hospital, Denmark.

**Fig. 1:**
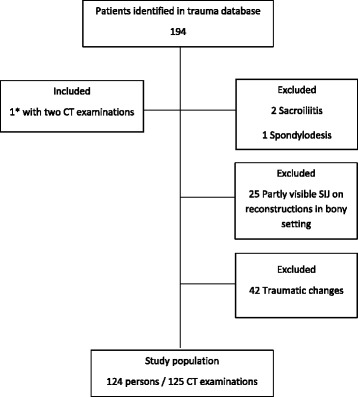
Flowchart of study population. * 1 girl included with two CT examinations being 13 and 15 years old

The study population comprised patients selected from a local trauma database at our hospital between January 2012 and August 2016. A total of 194 juvenile patients met our trauma team activation criteria according to the international ABCDE-system having a score ≥ 2 [17] and the indication for total-body CT used by Treskes et al. in the REACT-2 trial [18]. They all underwent CT scanning encompassing the head, neck, chest, abdomen and pelvis. Inclusion criteria to our study were: Age below 18 years and no CT detectable traumatic changes in the spine, pelvis, abdomen and chest. Exclusion criteria: Traumatic skeletal and/or organ lesions in the chest, abdomen, pelvis and spine, changes in the SIJ suggestive of sacroiliitis, previous spine or pelvic surgery, CT reconstructions in a bony setting not including the whole SIJ (Fig. 1). In total, 124 patients aged 9 months – <18 years (mean age 11 years 8 months), 57 girls and 67 boys, were included in our study. One girl was examined twice: at the age of 13 years and at the age of 15 years.

### CT examination

All patients were examined in supine position using a 64-slice Philips Brilliance CT scanner (USA, Cleveland), according to the standard department guidelines. A post-contrast CT examination of the thorax, abdomen and pelvis was performed using a beam collimation of 0.625 mm, slice thickness of 2 mm, increment 1 mm, kV 100–140 and mAs 100–350 appropriately adjusted for patient size and shape. Axial spine reformats, including the SIJ, with a slice thickness of 1 mm and an increment of 0.5 mm, using a bony reconstruction filter D, were routinely performed and post-processed at a Philips IntelliSpace workstation with software version 6.0.4.02700 for reconstructions of the SIJ. Axial, semi-axial and semi-coronal 2 mm thick contiguous CT slices covering the entire SIJ were used for evaluation of the SIJ.

### Evaluation of CT examination

**Fig. 2:**
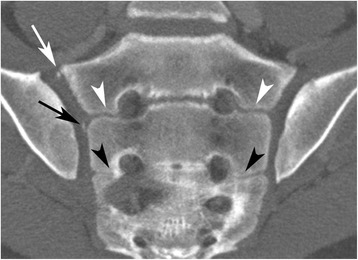
Fusion between sacral segments S1-S3 in a 14-year-old boy. Semi-coronal slice. Open space (no fusion) at the level S1/S2 – white arrowhead; partial fusion at the level S2/S3 – black arrowhead. Note also ossified nucleus antero-superior at S1 (white arrow) and lateral to the intervertebral space S1/S2 (black arrow) on the right side

**Fig. 3:**
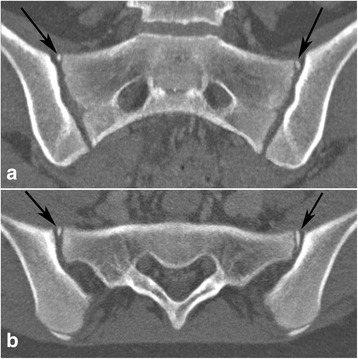
Ossified nuclei antero-superior at S1 in a 13-year-old girl. Bilateral ossified nuclei antero-superior at S1 - black arrows. **a** - semi-coronal and (**b**) - semi-axial slice

**Fig. 4:**
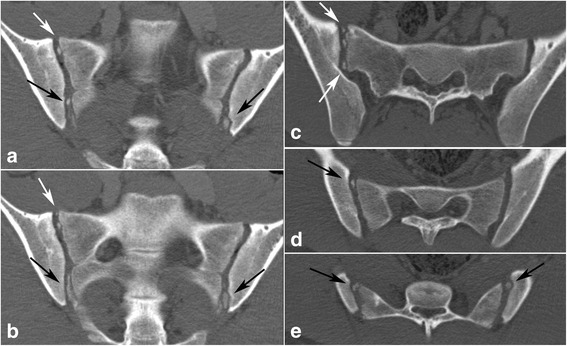
Multiple (≥ three) ossified nuclei in the joint space in a 16-year-old boy. Left images labeled (**a**), (**b**) – semi-coronal slices; right semi-axial images marked by (**c**) at the level of S1 and (**d**), (**e**) at the level of S2. White arrows – ossified nuclei lateral to the first sacral segment; black arrows – ossified nuclei lateral to the second sacral segment

**Fig. 5:**
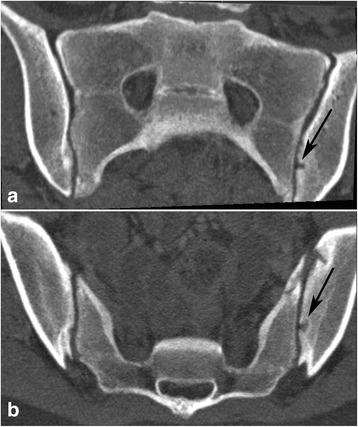
Joint facets defects ≥3 mm. 14-year-old girl with joint facets defect larger than 3 mm in the left iliac surface - black arrow; (**a**) - semi-coronal and (**b**) - semi-axial slice

The following anatomical features were recorded: 1) Intersegmental fusion of the sacral vertebral segments 1–3 (S1-S3) (Fig. 2), 2) ossified nuclei antero-superior at S1 (Fig. 3), 3) ossified nuclei lateral to the intersegmental spaces at S1/S2 and S2/S3 (Fig. 2), 4) ossified nuclei lateral to vertebral segment S1 and S2, respectively (Fig. 4), and 5) joint facet defects larger than 3 mm (Fig. 5).

Intersegmental fusion of sacral vertebrae S1 - S3 was characterized as absent when no osseous bridging was observed in the intersegmental space on all coronal slices. Partial fusion was reported in case of ossification in intersegmental spaces and complete fusion at the time of no detectable space between the sacral segments.

All 125 CT examinations of the SIJ were evaluated by a senior musculoskeletal radiologist (AZ). The reliability of the evaluations was assessed based on an independently evaluation of 48 examinations/96 SIJ by another senior radiologist (AGJ). Inter-observer agreement was based on these results.

### Statistical analysis

The frequency of segmental fusion and occurrence of ossified nuclei were analyzed descriptively in relation to age and gender. Gender related developmental differences were analyzed using Pearson’s chi-squared test or Fisher exact test. A level of *p* < 0.05 was considered significant.

Inter-observer variation regarding nuclei in the 96 joint spaces was assessed by Bland-Altman analysis using plots, bias and 95% confidence interval, and disagreements were subsequently analyzed to obtain consensus.

## Results

The two radiologists agreed on intersegmental fusion except in two (4%) of the 48 individuals and disagreed on joint facet defects in three of the 192 (1,5%) articular surfaces. They agreed on the number of nuclei in the joint space in 85 of the 96 (88%) joints. The disagreement regarding the number of nuclei was low with a bias of 0.1 and a 95% confidence interval of 0.0–0.2.

Results of the age and gender distribution of intersegmental fusion of S1-S3, occurrence of ossified nuclei antero-superior at S1 and in the SIJ space, and joint facet defects are shown in Table 1.

**Table 1:** Age and gender distribution of intersegmental fusion S1- S3, ossified nuclei, and joint facet defects

Age, years		<12		12- < 13		13- < 14		14- < 15		15- < 16		16- < 17		17- < 18	
Number of persons		Girls	Boys	Girls	Boys	Girls	Boys	Girls	Boys	Girls	Boys	Girls	Boys	Girls	Boys
		*n* = 27	n = 27	*n* = 3	*n* = 7	*n* = 6	*n* = 7	*n* = 8	*n* = 8	*n* = 4	*n* = 7	*n* = 4	*n* = 3	*n* = 6	*n* = 8
S1/S2 fusion: no		20	17	0	1	0	3	0	1	0	0	0	0	0	0
partial		6^a^	10^b^	3	6	2	4	3	7	2	4	0	1	1	4
complete		1^c^	0	0	0	4	0	5	0	2	3	4	2	5	4
S2/S3 fusion: no		20	24	0	1	1	2	1	2	0	0	0	0	0	1
partial		7^d^	3^e^	3	6	1	5	5	6	1	4	1	1	1	3
complete		0	0	0	0	4	0	2	0	3	3	3	2	5	4
Nuclei															
Ant/sup S1	bilateral	0	0	1	0	3	1	8	5	3	5	2	3	1	3
	unilateral	2^f^	0	0	0	1	1	0	2	0	1	0	0	1	0
Lat ISP S1/S2	bilateral	0	0	1	0	2	1	3	3	0	3	2	1	1	2
	unilateral	0	0	0	0	0	0	1	1	3	2	0	0	0	2
Lat ISP S2/S3	bilateral	0	0	0	0	0	0	4	1	1	0	2	3	1	1
	unilateral	0	0	0	0	0	0	0	1	0	1	0	0	0	0
Lat S1	bilateral	0	0	0	0	2	1	3	3	1	4	1	2	0	2
	unilateral	1^g^	0	1	0	0	0	1	1	2	2	0	0	1	0
Lat S2	bilateral	0	0	0	0	1	0	1	0	0	0	0	1	0	0
	unilateral	0	0	0	0	0	0	0	1	0	0	0	0	0	1
Joint facet defects															
bilateral		0	0	0	0	0	1	0	0	0	0	0	0	0	0
unilateral		1^h^	3^i^	1	3	3	1	2	2	1	1	1	0	1	0

^a^Occurrence at age 9 and 11 years, respectively; ^b^ Occurrence at age 6, 7, 9, 10 and 11, respectively; ^c^Occurrence at age 7 years; ^d^ Occurrence at age 9, 10 and 11 years, respectively; ^e^ Occurrence at age 10 and 11, respectively; ^f^ Occurrence at age 10 and 11 years, respectively; ^g^ Occurrence at age 11 years; ^h^Occurrence at age 11 years; ^i^Occurrence at age 7 and 8 years, respectively

Fusion of S1/S2 intersegmental space was not seen before the age of 6 years, and the frequency increased with age. Complete fusion was seen in one girl at 7 years and 10 months but predominantly occurred in girls after the age of 13; however, with remnants of space in one girl aged >16 years. Complete fusion was less common in boys occurring significantly less frequently in boys ≥14 years than in girls in the same age range (*X* ^2^ = 6.94, *p* = 0.008).

Fusion of the intersegmental space S2/S3 started at the age of 9 years, but there were remnants of space in both genders up to the age of 18 years. Complete fusion between S2 and S3 was observed 2 years earlier in girls than in boys, occurring in four of six girls aged 13–14 years and in three of seven boys aged 15–16 years, though not statistically significant.

Nuclei antero-superior at S1 and in the joint space rarely occurred before the age of 13 years, observed in three girls; two of them had both nuclei antero-superior at S1 and in the join space (in a 11-year-old girl lateral to S1 and in a 12-year-old girl lateral to S1 and at the level S1/S2, respectively). In individuals aged ≥13 years ossified nuclei at or in the joint space were frequent, occurring in 21 of 28 girls (75%) and in 26 of 33 boys (79%).

The antero-superior area at S1 was the most frequent location of ossified nuclei in individuals ≥13 years old, observed in 19 of 28 (68%) girls and 21 of 33 (64%) boys. The nuclei often occurred bilaterally and were visualized on both coronal and axial slices (Fig. 3). Nuclei at this location were first observed at the age of 10 years in a girl but were not seen in boys before the age of 13 years. Although they predominantly occurred in the age range 14 - ≤17 years, nuclei at this location were observed in two girls and three boys in the age range 17 - <18 years.

Intraarticular localization of ossified nuclei was seen in 18 of 28 (64%) girls and in 20 of 33 (60%) boys >13 years. In total, 25 of 61 persons (41%) aged ≥13 years had multiple (≥ three) ossified nuclei in the joint space, observed in 11 (39%) girls and 14 (42%) boys, respectively. Persistence of ≥ three nuclei located intraarticularly was observed in one girl and three boys ≥17 years old.

Joint facet defects over 3 mm were observed in 21 children (17%), 10 girls and 11 boys, occurring in all age groups. Bilateral defects occurred in one boy who had a total of three lesions (Fig. 5). Thirteen of the defects were localized to the iliac joint facet and ten to the sacral joint facet.

## Discussion

The present study highlights the complex osseous anatomy of juvenile SIJs during skeletal growth. There is a frequent occurrence of ossified nuclei antero-lateral to S1 or in the joint space in children and adolescents over 13 years (77% of individuals ≥13 years in current study). The occurrence of joint facet defects over 3 mm was observed in all age groups, but in 21% of individuals ≥13 years. Both ossified nuclei and joint facet defects could be challenging for radiologists or rheumatologists assessing MRI regarding inflammatory changes as growth related changes may simulate inflammatory lesions.

Computed tomography of juvenile SIJs is rarely use in the diagnosis of sacroiliitis due to the exposure to ionizing radiation. Review of the literature resulted in one study where 25 children/adolescents were examined by CT for unknown reasons. A total of seven individuals aged 15–19 years had ossified nuclei in the SIJ, all apparently occurring at the first sacral segment. The age of the remaining individuals in the study was not stated [16]. Another age range than in the present study may have influenced the lower frequency of nuclei compared to our detection of ossified nuclei in 50 of 125 (40%) individuals including nuclei in the joint space in 40 (32%) individuals. In the current study, ossified nuclei were observed 5 years earlier than in the study by Götz et al. [16] with the occurrence of ossified nuclei antero-lateral at S1 in one 10-year-old girl. We also detected nuclei more distally located in the joint space. Such nuclei were not detected by Götz et al. at CT, but their histological analyses of three autopsy specimens revealed the presence of nuclei at the level of the second and third sacral segment. It is therefore possible that the different scan techniques may influence results. The present use of 2 mm slices in three different reconstruction planes ought to increase the detectability of nuclei compared to axial 4–8 mm slices used in the study by Götz et al. [16].

The SIJ anatomy has also been evaluated by MRI [6]. Based on 114 individuals aged 8–17 years, findings regarding intersegmental fusion were rather similar to our observations with fusion occurring earlier in girls than in boys. Apophyses/nuclei lateral to S1 or at the intersegmental space S1/S2 were also observed between the age of 9 to 17 years. This is in accordance with the present findings except that Bollow et al. found complete ossification and apparently fusion of the nuclei at S1 in the 25 oldest children, mean age 15.9 ± 1.3 years. In the present study persistence of nuclei was also observed in the age group 17- < 18 years, in both girls and boys. The difference is probably due to a better delineation of minor osseous structures by CT than by MRI.

Growth-related changes can lead to false positive MRI results regarding both activity and structural lesions. Recent findings have revealed a limited specificity of BME alone to define a positive MRI for SpA in both children/adolescents and adults [19, 20]. Subtle non-specific BME at the SIJ suggestive of sacroiliitis was reported in 20% of a juvenile control group as well as in 21% of adult patients with low back pain (LBP) [20, 21]. The causes of subchondral non–inflammatory BME at the SIJ in juvenile subjects have not yet been analyzed. However, the high prevalence of ossified nuclei in the joint space in children and adolescents over 13 years could be the cause of the observed BME in 20% of the juvenile control subjects with a mean age ± SD of 15;1 ± 2;3 years [20]. An increased bone turn-over during ossification of cartilage-preformed bone could be a possible explanation. The ossified nuclei detected in the current study are probably located in the sacral cartilage, as shown histopathologically in three autopsy specimens [16]. The process resulting in fusion of the ossified nuclei to the main ossified sacral segment thus corresponds to the growth process occurring as part of epiphyseal cartilage fusion in tubular bones where the process is accompanied by BME. It has to our knowledge not been evaluated whether or not BME in healthy juvenile persons predominantly occur in the sacrum.

The presence of erosions was found to be a rather specific MRI feature for sacroiliitis in juvenile SpA patients, as it was reported to be present in 58–96% of patients [8, 20]. However, erosions by MRI of the SIJ were reported in 9% of a juvenile control group as well as in 11% of adults with non-inflammatory LBP [20, 21]. Defining erosion by MRI may be difficult due to a poor visualization of cortical bone in the widely used T1-weighted sequences and a high variability of the appearance of erosions. This is supported by moderate inter-observer agreement in juvenile individuals as well as in adults [20, 22]. Several studies evaluating the value of CT in the diagnosis of sacroiliitis in adults have shown higher specificity of CT for detection of erosions and subchondral sclerosis compared to radiography and the commonly used T1-weighted MR-images [11–13, 23–25]. Similar analyses have not been performed in juvenile populations to avoid radiation exposure by CT. Radiation disadvantages by CT have also been a limiting factor for assessing CT morphology in the healthy adult population. However, a study of 45 asymptomatic adults revealed bilateral erosions in only one case, indicating erosions to be rather specific signs of sacroiliitis [26]. In our study, irregularity of the osseous margins of the iliac and/or sacral articular surface with articular defects over 3 mm occurred in 17% and may simulate erosions by MRI.

One of the strengths of the current study is the wide age distribution illustrating the anatomical variety of the juvenile SIJ. Another strength is a uniform study recruitment and the standardized CT technique including reconstructions.

Some limitations need to be considered. The number of persons included in the analysis was rather small, resulting in few representatives among girls and boys in some of the age groups. Thus, our results must be interpreted with some caution. Only one reader evaluated all SIJ examinations, but the inter-observer agreement was good.

Knowledge of osseous anatomy at the SIJ is crucial to make a correct early diagnosis in children/adolescents, especially considering the appearance of structural changes in the early phase of SpA [27–29]. Further studies in a larger population and analyses of the correlation between CT and MRI appearances of structural findings in SIJ would be optimal but probably not feasible. A prospective study with MRI supplementing a CT examination performed on a clinical indication such as suspicious traumatic changes can be a possibility, though difficult to perform. However, the results of the current study could be used to define the anatomy of the SIJ by MRI in clinical practice. The possible presence of subchondral BME in relation to ossified nuclei in the articular cartilage needs to be investigated. An evaluation of SIJ anatomy in healthy juvenile persons using dedicated cartilage sequences and high-resolution 3 T image quality can potentially visualize the complex anatomy and explain BME distribution on concomitant STIR or T2- weighted fat suppressed sequences.

## Conclusions

Normal osseous SIJ structures in children and adolescents vary considerably. Intraarticular ossified nuclei are frequent after the age of 13 years and can persist up to the age of 18 years in both genders. Attention to normal anatomical structures during growth may help to avoid false positive MRI findings.

## Abbreviations

ASAS: Assessment of SpondyloArthritis International Society
BME: Bone marrow edema
CT: Computed tomography
JSpA: Juvenile Spondyloarthritis
LBP: Low back pain
MRI: Magnetic resonance imaging
SIJ: Sacroiliac joints
SpA: Spondyloarthritis
